# Detecting Aquatic Vegetation Changes in Taihu Lake, China Using Multi-temporal Satellite Imagery

**DOI:** 10.3390/s8063988

**Published:** 2008-06-25

**Authors:** Ronghua Ma, Hongtao Duan, Xiaohong Gu, Shouxuan Zhang

**Affiliations:** State Key Laboratory of Lake Science and Environment, Nanjing Institute of Geography and Limnology, Chinese Academy of Sciences, Nanjing, PO Box 210008, People's Republic of China

**Keywords:** Aquatic vegetation, Taihu Lake, Decision tree, Biomass, Remote sensing

## Abstract

We have measured the water quality and bio-optical parameters of 94 samples from Taihu Lake *in situ* and/or in the lab between June 10-18, 2007. A transparency-assisted decision tree was developed to more accurately divide the aquatic vegetation zone into a floating vegetation-dominated zone and a submerged vegetation-dominated zone, whose respective present biomass retrieval models were easily developed with an empirical approach because of the quasi-concurrence of ground field investigations with the satellite sensor flight over the lake. The significant quantitative relationships between the vegetation index NDVI (Normalized Difference Vegetation Index) of different images at different times were used to help develop the past biomass retrieval model on the basis of the present developed model. In Taihu Lake, the total covering area of aquatic vegetations decreased from 454.6 km^2^ in 2001 to 364.1 km^2^ in 2007. Correspondingly, the total biomass decreased from 489,000 tons in 2001 to 406,000 tons in 2007, suggesting that a great change in the ecological environment has been taking place in Taihu Lake over this period.

## 1. Introduction

Aquatic vegetation, generally existing in the shallow near-shore area, is a key component of lake ecosystems. This vegetation provides food, shelter and breeding habitats for aquatic animals like invertebrates, fish and wading birds, and helps maintain the balance of the lake ecosystem. In addition, it also plays an important role in maintaining a clean lake water quality by stabilizing sediments and providing a substrate for periphyton that actively removes nitrogen and phosphorus from the water column. At times and locations where submerged vegetation is very abundant, water is clear, and phytoplankton blooms are rare. It almost becomes a token indicator to determine whether the water quality can be expected to be good or not. The spatial extent and biomass of aquatic vegetation, especially submerged aquatic vegetation, are expected to increase significantly as a result of regional restoration programs, so the past and present status of a regional biomass and distribution of aquatic vegetation are necessary for the monitoring, management and understanding of shallow aquatic ecosystems, which need baseline measurements to document their extent and condition. Determining the vegetation cover and biomass is difficult, however, both on small and large spatial scales because of the spatial heterogeneity of these communities [1]. It has been indicated that aquatic vegetation yields spectrally distinct signals governed by the density of the vegetation, the openness of the canopy and the amounts, forms and orientations of the leaves [2-5]. Since traditional quantitative ground investigations on the scale of a whole lake are laborious, remote sensing methods are increasingly being used for mapping aquatic vegetation and estimating their distribution and biomass [6-9].

Multi-temporal remote sensing data can give valuable information about changes that have taken place in a given area [8]. It is also well suited to identify both emergent and submerged vegetation [1, 10]. Aerial photographs were commonly used for mapping aquatic vegetation in the earlier period [3, 5, 7], but with rapid development of satellite sensors, satellite multi-spectral scanner data are widely used at present. Landsat TM (Thematic Mapper) has been proven very effective for aquatic vegetation distribution and biomass mapping applications [11, 12], however, more detailed spatial monitoring is still not possible due to its somewhat coarse spatial resolution [13]. IKONOS image, with enough small pixel size, may be used to make fine-level habitat discrimination by making full use of within-habitat textural information in a supervised classification, which significantly improved the thematic map accuracy compared with Landsat TM image; however, it cannot spectrally resolve changes in community structure [14]. The use of Hymap images with hyper-spectral resolution has been attempted to provide a spectral discrimination of submerged macrophytes [15, 16]. Supervised classification [11, 14] or visual interpretation [1] is a common method for discrimination of emerged and submerged vegetation, both of which have significantly different spectral features in the visible and near-infrared wavelengths. Ackleson and Klemas [17] demonstrated that the accuracy of results could be increased significantly when a prior knowledge of water depth was added to the classification crteria because of the close correlation between the distribution of aquatic vegetation and water depth [18]. The spectral confusion between submerged vegetation canopy density and deep water could be solved significantly. Malthus and George [19] demonstrated that a combination of band 3 (520-600 nm), band 7 (760-900 nm), and band 8 (910-1050 nm) data from the Daedalus Airborne Thematic Mapper could discriminate between different macrophyte growth forms. The DN (Digital Number) value, a sum of contributions due to atmosphere, water column and bottom, has been most commonly used to estimate vegetation biomass with an empirical linear or non-linear fitting model [9]. If the influence of the water column could be removed from the remotely sensed images, the potential of optical remote sensing in littoral applications would be extended and the accuracy of classification and biomass retrieval would be improved to a certain extent [20, 21]. As optical field equipments have become available, the bio-optical model has appeared and is being widely used, which make it possible to accurately remove the contribution due to water column [22, 23] and then to separate between the signatures coming from water column and bottom.

This paper aims to present an approach to estimate the past biomass of aquatic vegetation in a shallow inland lake using the historical satellite imageries without any corresponding field investigations, based on the present satellite imagery provided along with concurrent or quasi-concurrent ground field investigations, and then to determine the historical change of the aquatic vegetation in Taihu Lake. We first present some general information about Taihu Lake and the field investigation, including instruments and sampling methods, and satellite imageries together with its preprocessing method. Then we describe the method for developing the quantitative estimation model for aquatic vegetation in Taihu Lake. Finally, some conclusions are drawn on the basis of the full discussion.

## 2. Study area

**Figure 1.**
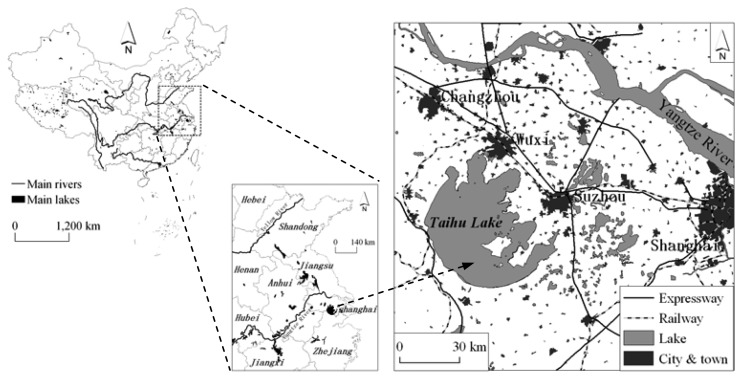
Location of Taihu Lake in China.

Taihu Lake, a large shallow lake with an average depth of 1.9 m (max. 2.6 m), and covering an area of 2427.8 km^2^ (including 51 islands), is one of the five largest fresh-water lakes of China and is located in the core of the Yangtze Delta in the lower reaches of Yangtze River, in East China, an area that has a developed economy (Figure 1). Unfortunately, in recent years, water quality has deteriorated and cyanobacteria blooms have covered large areas of the lake every summer since the 1990s. There exist four types of aquatic vegetation: (a) emerged vegetation, including *Phragnites communis*, *Typha angustifolia*, *Zizania latifolia* and so on, (b) submerged vegetation, including *Ceratophyllum demersum*, *Vallisneria spiralis*, P. *malaianus* and so on, (c) floating vegetation, including *Eichhornia crassipes*, *Lemna minor* and so on, and (d) floating-leaved vegetation, including *Nyphar pumilum*, *Nymphaea tetragona* and so on. They are mainly distributed in the several bays of the east and southeast and in the south littoral zones, where the water is clear and cyanobacteria blooms are rare.

## 3. Material

### 3.1 Field data

**Figure 2.**
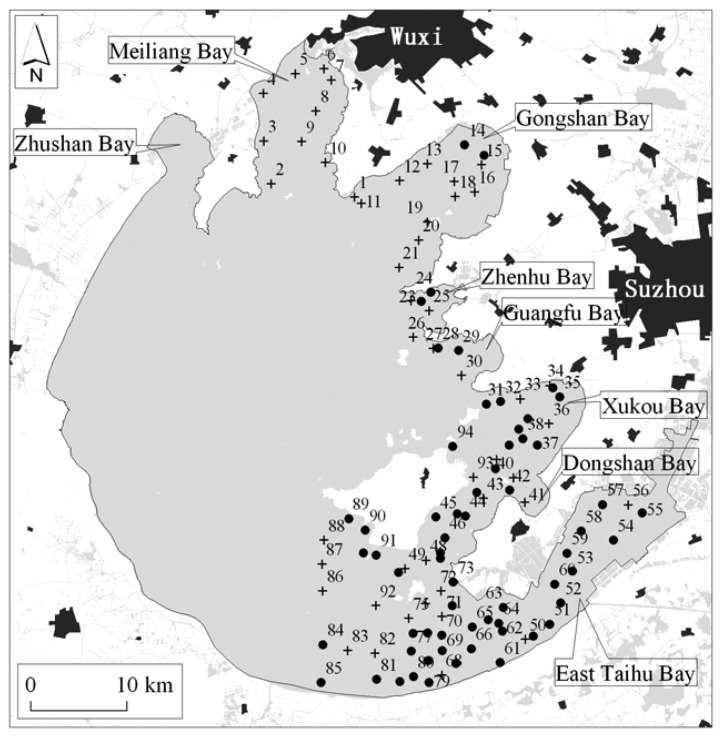
Locations of field samples investigated on 10-19 June 2007; there is aquatic vegetation in the black circle samples and there isn't in the cross samples.

The campaign was carried out along the preset transects for 94 samples on 10-18 June 2007 (Figure 2), which were positioned by differential GPS (Global Positioning System, Trimble Ltd., USA) with a positioning precision of 2-5 m. Before the field investigations, the sampling locations were preset according to the prior knowledge of the aquatic vegetation distribution with help of a 1:50,000 scale digital topographic map. This prior knowledge comes from the previous six field investigations of the spatial distribution of aquatic vegetation for 95 samples in October 2006, 133 samples in May 2006, 27 samples in October 2005, 80 samples in June 2005, 25 samples in July 2003, and 78 samples in September 2002, respectively.

The remote sensing reflectance and the backscattering coefficient, respectively, were measured *in situ* with a dual channel spectrometer FieldSpec 931 (ASD Ltd.) and a HydroScat-6 Spectral Backscattering Sensor (HS-6, HOBI Lab Inc.) mounted at six wavelengths (centered at 442, 488, 532, 589, 676 and 852 nm, respectively). The instruments, methods of measurement and data processing are the same as in Ma *et al.* [22, 23]. Water samples were collected from the surface to about 30 cm below in the vertical direction with a standard 21 polyethylene water-fetching instrument immediately after measuring the spectra. They were then held in a freezer half filled with ice bags for preservation for approximately 4 h every afternoon, and then returned to the laboratory for concentration and absorption measurements. The transparency was measured with a Secchi disk at the shade side of the boat. We employed the boat-based rake method as described by Rodusky *et al.* [19], with an oyster-tongs-like rake apparatus operated from a boat, to collect aquatic vegetation, which were put on the deck or into a plastic basin for flushing with local lake waters in order to remove the attached mud. We also measured the reflectance of aquatic vegetation, mud and the mix of vegetation and mud collected from the bottom using the same spectrometer in the wavelength range of 350-1050 nm. All the clean vegetation at one site, including leaves brushed off, was put into a plastic bag with a serial number. The plastic bag was then held in a freezer half filled with ice bags for preservation and together with water samples, returned to the laboratory for species classification and biomass measurements. In addition, relative species depth (surface, sub-surface, and deep) within the water column was measured at each sample site, and the sampling time was also recorded point by point.

In the lab, the concentrations of DOC (Dissolved Organic Color, *C*_DOC_), chlorophyll-a (*C*_CHL_), and SPM (Suspended Particulate Matter, *C*_SPM_) were measured according to the investigation criteria for Chinese lakes [24]. We also measured the absorptions of phytoplankton pigment (*a*_ph_), non-algal particulate (*a*_d_), and CDOM (Colored Dissolved Organic Matter, *a*_g_) according to the NASA SeaWiFS protocols described by Mueller *et al.* [25]. Details about the concentration and absorption measurements can be found in Ma *et al.* [22, 23]. The absorption and backscattering due to pure water, *a*_w_ and *b*_bw_, refers to the definitions of Pope and Fry [26].

### 3.2 Satellite data

IRS P6 (Indian Remote Sensing Satellite Resourcesat-1) LISS-3 (Linear Imaging Self-Scanning Sensor-3) image with a spatial resolution of 23.5 m, mounted at three bands in the range from 520 nm to 860 nm and one band from 1550 nm to 1700 nm was first used to develop the basic and primary model for biomass estimation of aquatic vegetation in Taihu Lake. The imaging date selected for this research was 15 June 2007. In Taihu Lake, based on our experience, aquatic vegetation grows slowly before July every year, so we may reasonably assume that the vegetation changes little in the short time range of our field investigation and we may consider that the ground field investigation and the LISS-3 sensor flight over the lake are almost concurrent. In order to establish the annual change of aquatic vegetation in Taihu Lake, we also employed some Landsat TM images, with a spatial resolution of 30 m, with hardly any cloud cover and almost the same date, respectively, acquired on 26 July 2001, 13 July 2002, and 26 July 2004.

## 4. Methods

### 4.1 Image preprocessing

Two methods were employed to remove the contributions due to the atmosphere in order to develop the two different biomass estimation models, viz. an empirical model and an analytical model. One is referred to an assumption that the water-leaving radiance in the near-infrared wavelength is zero, which is commonly used in the case one water color remote sensing. This method is simply approximate and aims to empirically estimate the aquatic vegetation biomass. A pixel with the lowest DN value exists in each of bands, which is assumed to be fully due to atmosphere. The pixel is first identified if it meets the following two requirements: (a) it is in optically deep clear water, or in the shallow clear water but the bottom is mud without concomitant vegetation, and (b) its DN value is the lowest in the corresponding band mounted on the LISS-3 or TM sensor. Then we may subtract the contribution due to atmosphere from total signals to leave only the signal due to water column and bottom. The steps mentioned above are repeated until all the bands mounted at the sensor are finished. The other is complexly approximate, referring to the methods for terrestrial remote sensing. Here, FLAASH (Fast Line-of-sight Atmospheric Analysis of Spectral Hypercubes), an atmospheric correction code based on the MODTRAN (MODerate resolution atmospheric TRANsmission) 4 radiative transfer model, is used to convert LISS-3 and TM sensor radiance data to apparent reflectance, aiming to develop an analytical approach to estimate aquatic biomass. Subsequently, a geometrical correction was done using a 1:50,000 scale topographic map (spheroid: Krasovsky 1940, projection: Gauss-Krüger). The RMSE (Root Mean Square Error) for each of the images was maintained at less than 0.5 pixels. Finally, the Taihu Lake boundary from the same topographic map is used as a binary mask to create a water body-only image.

### 4.2 Image classification

**Figure 3.**
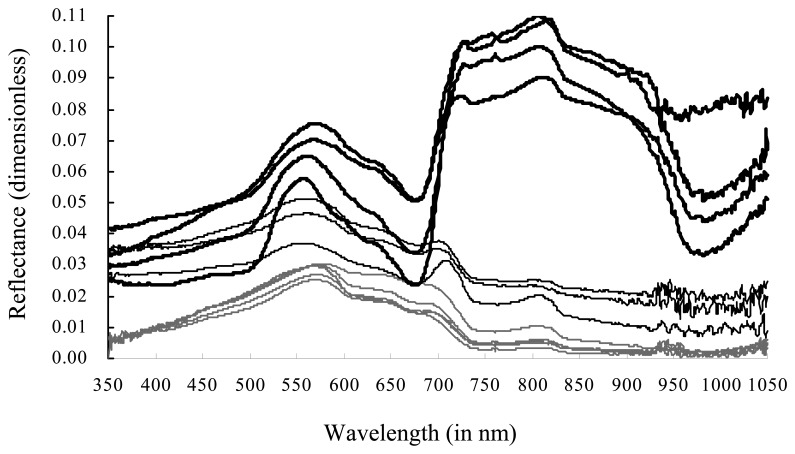
Comparisons between remote sensing reflectance only due to water (the thin gray curves), reflectance due to Type one (the thick black curves), reflectance due to Type two (the thin black curves), all of which were measured *in situ*. The *x*-axes is wavelength (in nm), and the *y*-axes is reflectance (dimensionless).

The boundary between various classes of emergent, floating, floating-leaved and submerged vegetation, whose biomass in unit area have significant differences, is not distinct, as these communities merge into one another. To make it suitable for the remote sensing application and then to make the biomass estimation precision as high as possible, the aquatic vegetation zone is divided into two types: (a) Type one, with a characteristic of the leaf standing above the water surface, including the floating-leaved and emergent vegetation, named a floating vegetation-dominated zone, and (b) Type two, with the characteristic feature of the whole plant from canopy to root being submerged beneath the water surface, including submerged and floating vegetation, named as the submerged vegetation-dominated zone. Spectral features due to Type one in the range of red and near-infrared wavelengths are greatly different from the ones due to Type two (Figure 3). The two types of vegetations are easily separable in optical satellite remote sensing images.

Prior to separating the aquatic vegetation into the two types, it is of vital importance to first separate them from lake water. The submerged vegetation-dominated hydrophytes, however, commonly show a similar spectral feature with lake waters containing many phytoplankton or suspended particulate matter (Figure 3).

It is not easy to separate them only depending on their spectral characteristics. So we add the prior knowledge, including water depth and transparency, into the classification process to develop a decision tree as the following steps: (a) separate the vegetation and quasi-vegetation from lake water with help of the prior knowledge about relationships between depth, transparency and vegetation distribution, (b) to calculate vegetation index *RVI* (Ratio Vegetation Index, a ratio of reflectance in the near-infrared waveband to in the red wavelength) and *NDVI* (Normalized Difference Vegetation Index, a ratio of the reflectance difference between in the near-infrared and red wavebands to the sum of the reflectance in the two wavebands), and then to remove Type one according to the correct threshold values of *RVI*, *NDVI* and *SDT* (Secchi Disk Transparency), (c) to change the threshold values of *RVI*, *NDVI* and *SDT* to remove the non-vegetation from vegetation and quasi-vegetation zone, and then only leave the Type two.

**Figure 4.**
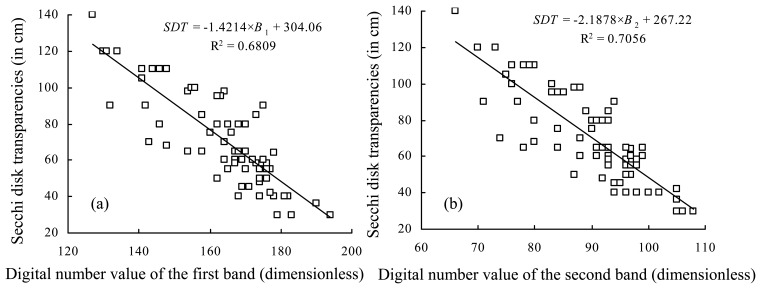
Relationships between the Secchi disk transparencies measured *in situ* and the atmosphere-corrected digital number values of: (a) the first band (*B*_1_) mounted at LISS-3 sensor, and (b) the second band (*B*_2_). Both of the *y*-axes are the Secchi disk transparencies (in cm), x-axes in (a) and (b), respectively, are *B*_1_ and *B*_2_ (in dimensionless).

The depth comes from a bottom topographic map with a scale of 1:50,000 mapped in 1998, and corrected *in situ* during this field investigation. The transparency may be retrieved from an empirical model given by Equation (1). There are good relationships, with determination coefficients (R^2^) of more than 0.68, between the Secchi disk transparencies measured *in situ* and the atmosphere-corrected DN values of the first (*B_1_*) and the second band (*B_2_*) mounted at LISS-3 sensor, respectively (Figure 4). However, there is a better relationship between the Secchi disk transparencies measured *in situ* and both of the atmosphere-corrected DN values of the former two bands mounted at LISS-3 sensor given as:

(1) $$\text{SDT}=279.330-0.708\times B_{1}-1.033\times B_{2}\;(R^{2}=0.82,\;\text{N}=70)$$

where *SDT* (in cm), R^2^, *B*_1_ and *B*_2_ are the same as mentioned above; N is the total sampling number, not including samples in the floating vegetation-dominated zone.

### 4.3 Biomass estimation

The submerged vegetation zone is optically shallow in Taihu Lake [22], so [27]:

(2) $$r_{rs}={r_{rs}}^{c}+{r_{rs}}^{B}\approx {r_{rs}}^{dp}\left(1-\exp\left\{-\left[\frac{1}{\cos(\theta_{w})}+{D_{u}}^{c}\right]\kappa H\right\}\right)+\frac{1}{\pi }\rho \exp\left\{-\left[\frac{1}{\cos(\theta_{w})}+{D_{u}}^{B}\right]\kappa H\right\}$$

where

(3) $$r_{rs}^{dp}\approx (0.084+0.170u)u$$

(4) $${D_{u}}^{c}\approx 1.03{\left(1+2.4u\right)}^{0.5}$$

(5) $${D_{u}}^{B}\approx 1.04{\left(1+5.4u\right)}^{0.5}$$

(6) $$u=b_{b}/(a+b_{b})$$

(7) $$\kappa =a+b_{b}$$

(8) $$R_{rs}\approx \frac{0.5r_{rs}}{1-1.5r_{rs}}$$

where *R_rs_* is remote sensing reflectance; *r_rs_* is the remote sensing reflectance just below the surface; *r*_rs_*^c^* and *r_rs_^B^* are the signals, respectively, due to water column and bottom; *θ_w_* is the subsurface solar zenith angle; *H* is the bottom depth; *a* is the total absorption coefficient, a sum of *a_ph_*, *a_d_*, *a_g_* and *a_w_*; *b_b_* is the total backscattering coefficient measured by HS-6.

**Figure 5.**
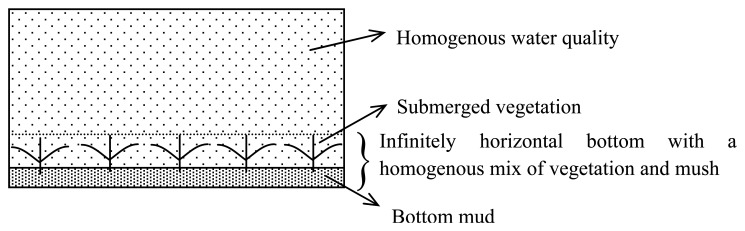
Sketch map of the hypothesis condition.

The FLAASH atmosphere-corrected signal was first converted to the signal just below the surface, which might be decomposed into the contributions due to water column and due to lake bottom using Equation (2) on the basis of the hypothesis conditions of Figure 5: (a) infinitely horizontal bottom with a homogenous mix of vegetation and mush with a Lambert property, and (b) homogenous water quality in vertical, so the contribution due to water column can be approximately removed by Equation (2) to leave the remote sensing reflectance only due to bottom. Then we developed the biomass estimation model by the relationship between the vegetation biomass and the remote sensing reflectance only due to bottom, however, which always was bad no matter what efforts we paid out. So here the semi-analytical approach is not suitable for aquatic vegetation biomass estimation in Taihu Lake. Subsequently, we have to attempt to develop an empirical model between the atmosphere-corrected DN value and the biomass measured in the field investigation.

In the LISS-3 image on 15 June 2007 good empirical relationships between the biomass in unit area of the two types of aquatic vegetation mentioned above, respectively, and *NDVI* and *SDT*, existed which can be described as:

(9) $$\{\begin{array}{ll} BM1_{\text{LISS}3}=2.760+4.081\times \text{NDV}I_{\text{LISS}3} & \left(\begin{array}{cc} {\text{R}}^{2}=0.623, & \text{N}=14 \end{array}\right)\\ BM2_{\text{LISS}3}=1.260-0.011\times \text{SDT}-2.415\times \text{NDV}I_{\text{LISS}3} & \left(\begin{array}{cc} {\text{R}}^{2}=0.782, & \text{N}=28 \end{array}\right) \end{array}$$

or

(10) $$\{\begin{array}{ll} BM1_{\text{LISS}3}=2.760+4.081\times \text{NDV}I_{\text{LISS}3} & \left(\begin{array}{cc} {\text{R}}^{2}=0.623, & \text{N}=14 \end{array}\right)\\ BM2_{\text{LISS}3}=0.137-3.542\times \text{NDV}I_{\text{LISS}3} & \left(\begin{array}{cc} {\text{R}}^{2}=0.697, & \text{N}=28 \end{array}\right) \end{array}$$

where *BM*1*_LISS3_* (in kg/m^2^), *BM*2*_LISS3_* (in kg/m^2^) and *NDVI_LISS3_*, respectively, are the vegetation biomass in unit area of Type one and Type two and the vegetation index *NDVI* from LISS-3 image on 15 June 2007; *SDT* (in cm) is transparency coming from Equation (1); R^2^ is the determination coefficient and N is the total samples in the floating vegetation-dominated or submerged vegetation-dominated zone. There was no corresponding biomass data from field investigations on 26 July 2001, 13 July 2002, and 26 July 2004 when the Landsat TM sensor was flying over the Taihu Lake. In order to approximately acquire the historical aquatic vegetation biomass at the corresponding time, these Landsat TM image pixels are firstly re-sampled into the same spatial resolution of 23.5 m as LISS-3 image pixel. And then we use the pure pixels with growing vegetations in the land, whose canopy is almost invariable between TM and LISS-3 images, to develop the quantitative *NDVI* relationships between the two images, so:

(11) $$\begin{array}{cc} \text{NDV}I_{\text{LISS}3}=-0.114+0.224\times \text{NDV}I_{TM2001} & \left(\begin{array}{cc} {\text{R}}^{2}=0.660, & \text{N}=40 \end{array}\right) \end{array}$$

(12) $$\begin{array}{cc} \text{NDV}I_{\text{LISS}3}=-0.083+2.269\times \text{NDV}I_{TM2002} & \left(\begin{array}{cc} {\text{R}}^{2}=0.561, & \text{N}=40 \end{array}\right) \end{array}$$

(13) $$\begin{array}{cc} \text{NDV}I_{\text{LISS}3}=-0.104+0.310\times \text{NDV}I_{TM2004} & \left(\begin{array}{cc} {\text{R}}^{2}=0.670, & \text{N}=40 \end{array}\right) \end{array}$$

where *NDVI_TM2001_*, *NDVI_TM2002_* and *NDVI_TM2004_* are the vegetation index *NDVI* from Landsat TM images, respectively, on 26 July 2001, 13 July 2002, 26 July 2004, R^2^ is the determination coefficient and N is the total sample number. It's a pity that the relationships of transparency between TM and LISS-3 images cannot be set up because of no transparency data measured *in situ* at the time when TM images was acquired. However, the water quality in those vegetation zones is hardly variational from 2001 to 2007 [28]. So we could, respectively, substitute Equation (11)-(13) into Equation (10), so:

(14) $$\{\begin{array}{c} BM1_{TM2001}=2.295+0.914\times \text{NDV}I_{TM2001}\\ BM2_{TM2001}=0.541-0.793\times \text{NDV}I_{TM2001} \end{array}$$

(15) $$\{\begin{array}{c} BM1_{TM2002}=2.421+1.098\times \text{NDV}I_{TM2002}\\ BM2_{TM2002}=0.431-0.953\times \text{NDV}I_{TM2002} \end{array}$$

(16) $$\{\begin{array}{c} BM1_{TM2004}=2.336+1.265\times \text{NDV}I_{TM2004}\\ BM2_{TM2004}=0.505-1.098\times \text{NDV}I_{TM2004} \end{array}$$

where *BM*1 and *BM*2 (in kg/m^2^), respectively, denote the vegetation biomass of Type one and Type two; the subscripts represent the sensor and the date when the image was acquired by the sensor (here 26 July 2001, 13 July 2002, 26 July 2004). Finally, the error matrix from the powerful *Accuracy Assessment* tool in the ERDAS 9.0 software was used to evaluate the remote sensing classification accuracy and then the estimation accuracy for vegetation biomass in 2007 was assessed by comparison of the estimated and the measured ones, based on which, the accuracies in 2001, 2002 and 2004 were estimated by the error transfer law commonly used in the field of surveying and mapping sciences [29].

## 5. Results

### 5.1 Field investigation

This investigation showed that aquatic vegetations with a wet biomass range from 0.11 to 3.98 kg/m^2^ and a standard deviation of 0.98 kg/m^2^ existed only at 42 sampling sites. The emerged vegetation mainly is *Phragmites communi*, located within a near-shore range of about 100 m. It is outside this biomass estimation. The remainder is growing in six clusters: *Potamogeton malaianus* cluster, *Nymphoides peltatum* cluster, *Nymphoides peltatum-Potamogeton malaianus* cluster, *Vallisneria natanus* cluster, *Vallisneria natanus-Elodea nuttalli* cluster, and *Najas marina* cluster, as in Table 1. They can be classified into two classes: (a) the floating vegetation-dominated hydrophyte, growing at 14 sampling sites, mainly including *Nymphoides peltatums*, and (b) the submerged vegetation-dominated hydrophyte, growing at 28 sampling sites, mainly including *Potamogeton malaianu*.

## 5.1 Remote sensing classification and estimation

**Figure 6.**
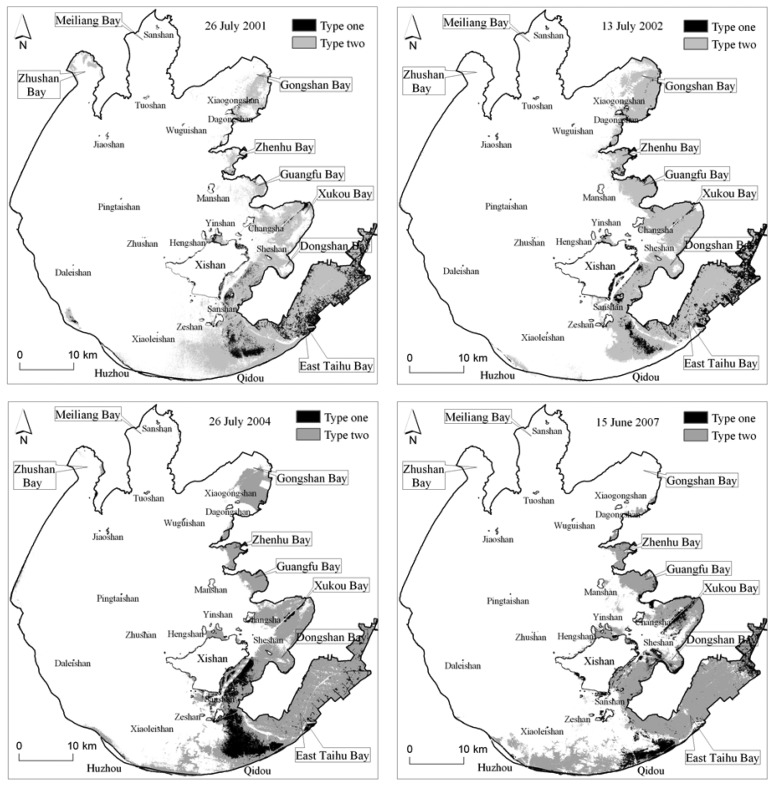
Spatial distribution of the aquatic vegetation except Phragmites communi on 26 July 2001, 13 July 2002, 26 July 2004, and 16 June 2007; Type one and Type two, respectively, are the floating vegetation-dominated hydrophyte, and the submerged vegetation-dominated hydrophyte.

The satellite image classification, illustrated in Figure 6 and Table 2, shows in Taihu Lake that the aquatic vegetation has undergone a great spatial change since 2001. The coverage area in both 2001 and 2002 were basically constant around 450 km^2^, and in 2004 increased up to 482 km^2^.

However in 2007, it decreased rapidly to 361 km^2^. The vegetation in Gongshan Bay, Zhenhu Bay, Guangfu Bay and Xukou Bay were spreading around in 2001-2002; however in 2002-2004, they began to move back towards to the bay interior or one side of the shore. Noteworthily, the vegetation in Gongshan Bay almost completely vanished according to the remote sensing investigations in 2007. In fact, the field investigation showed that in the north of Gongshan Bay there is only a little vegetation with a very thinly scattered distribution. The same situation as in Gongshan Bay in 2007 also happened in Zhushan Bay in 2002. Some degradation happened to a certain extent along the route from Xiaoleishan island to the mouth of East Taihu Bay in 2002. On the contrary, since 2004, some vegetation was growing near the shore at the south of Xishan island. In addition, we obviously observe that some vegetation has appeared along the southern littoral zone from Qidou to Huzhou since 2004, and that it was spreading towards to the north at the same time of extending towards to the west along the shore.

**Figure 7.**
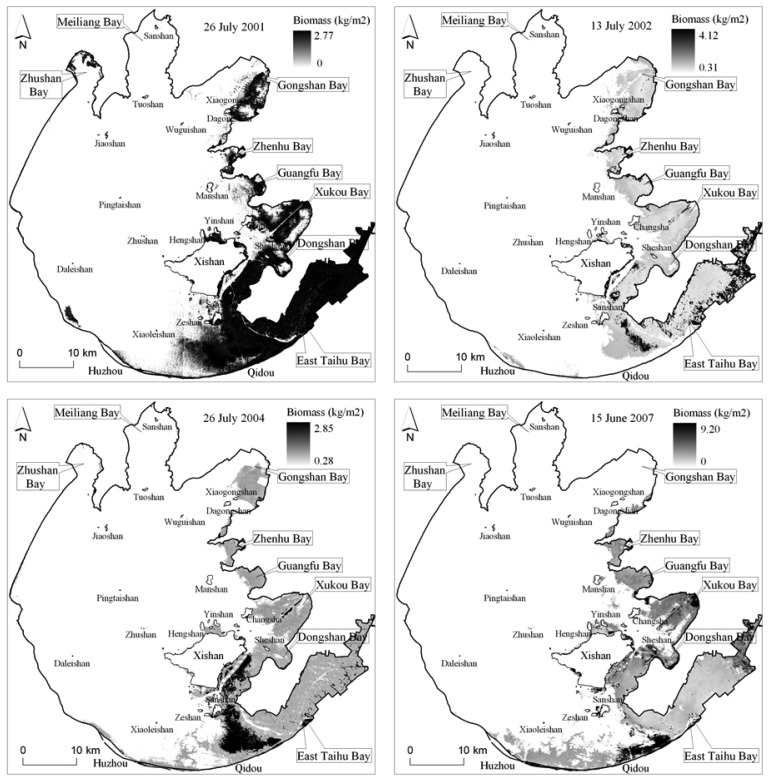
The satellite-estimated biomass of aquatic vegetation on 26 July 2001, 13 July 2002, 26 July 2004, and 16 June 2007.

The estimated vegetation biomass following the decision tree classification, illustrated as Figure 7 and Table 2, shows that the total biomass was basically stable about 480 thousand ton in 2001 and 2002, then increased up to 528 thousand ton in 2004, and then decreased rapidly up to 406 thousand ton in 2007 because of its covering area decreasing rapidly. However, in some special lake zone such as Zhenhu Bay and Guangfu Bay, the vegetation biomass increased significantly but its covering area only has a little difference, which shows that the vegetation has a more flourishing growth in these zones.

**Figure 8.**
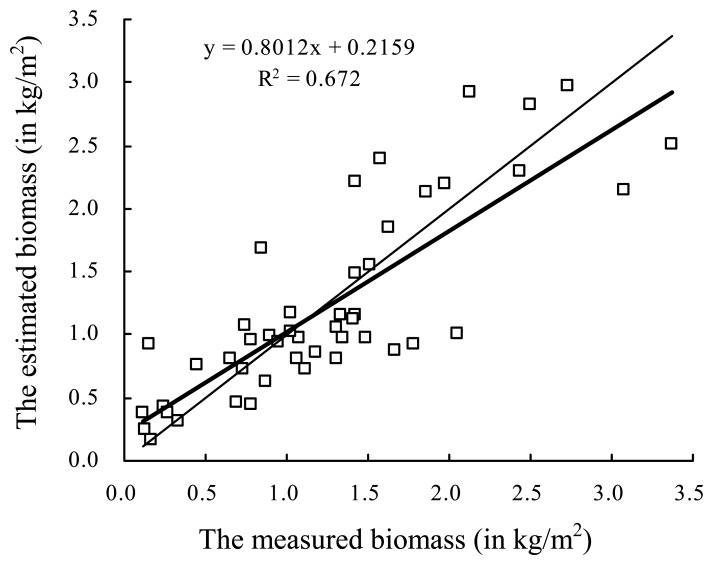
Scatter-map of biomass between the measured in situ (x-axis, in kg/m2) and the estimated by remote sensing (y-axis, in kg/m2). The thick line is the fitting curve by a least square method, and the thin is the 1:1 line.

The classification precisions are up to 90.8%, 88.9%, 90.2% and 89.2%, respectively, for LISS-3 image in 2007, TM images in 2001, 2002 and 2004. For the LISS-3 image in 2007, the maximum and minimum absolute errors of biomass estimation, respectively, are 2.60 kg/m^2^ and 0.001 kg/m^2^, and the maximum and minimum relative errors, respectively, are 287.03% and 0.10%, which were acquired by analysis and comparison between 46 sampling sits growing aquatic vegetations (Figure 8). The average absolute errors of Equation (11)-(13) are, respectively, 0.049, 0.048, and 0.044, and the corresponding average relative errors are, respectively, 34.64%, 35.49%, and 29.55%. The total biomass estimation error in 2007 is about 46.3 thousand ton, and the errors in 2004, 2002 and 2001 are, respectively, about 70.2, 67.5, and 64.4 thousand ton according to the error transfer law from Equation (11)-(13), through Equation (10), to Equation (14)-(16).

## 6. Discussion

It is difficult or impossible to accurately estimate the historical biomass of aquatic vegetation and then to discover its change between the past and the present if there was no field investigations and also no corresponding remote sensing image obtained at the same time. The present empirical estimation model could be developed on the basis of the field investigation and the concurrent or quasi-concurrent satellite imagery at the corresponding time, and then may be used to work out the past estimation model by rightly fusing the relationship between vegetation indexes from the present and past satellite imageries. Another approach is firstly to remove the contribution due to water column from the atmosphere-corrected signals through the radiative transfer in natural waters on the basis of several perfect assumptions. And then the remote sensing signal only due to the bottom was acquired and then used to develop the retrieval model of the submerged vegetation or submerged vegetation-dominated hydrophyte biomass. The actual situation, however, is a little far away from the perfect assumptions, which may result in a big error for removing the contribution due to water column. So it is not applicable and practicable very well.

Accurate separation of the aquatic vegetation from lake area is the first step to accurately estimate the biomass using remote sensing images. In this paper, the aquatic vegetation zone in Taihu Lake was divided into the floating vegetation-dominated and submerged vegetation-dominated hydrophyte zones, which only is a way to improve biomass estimation precision based on the characteristic of different species of aquatic vegetation growing in a cluster from a viewpoint of remote sensing. In Taihu Lake, both of the water depth and transparency in aquatic vegetation zone have a distinct difference with in other zones. The prior knowledge on depth and transparency can help discriminate the confusion between vegetation and non-vegetation zones and then increase their separability and classification accuracy to a certain ex0tent; in the same way, the estimated biomass, especially for Type one by Equation (9) is more accurate than by Equation (10). It's a pity, however, that no such prior knowledge about transparency was provided in the corresponding date when the Landsat TM images used in this study was acquired. Although the historical transparency could be estimated using the empirical model as Equation (1) by the Landsat TM images, the estimated result could not be verified because of no data measured *in situ*. So we left off the transparency data in the historical biomass estimation using the Landsat TM images.

Maybe it is more perfect and more accurate for the total biomass if we may implement biomass estimation of each species of aquatic vegetations, which, however, is very difficult in Taihu Lake. Firstly, the aquatic vegetation grows in a cluster including several species, so there hardly is a pure vegetation pixel in the satellite imagery with a coarse spatial resolution of about 20-30 m, where only a single species of aquatic vegetation grows. The spectral signal of vegetation in one pixel of satellite imagery comes from all the aquatic vegetations in the range of field-of-view angle. Additionally, most of multi-spectral sensors have a coarse spectral resolution of about 60-90 nm in the visible wavelength, which results in a difficulty to differentiate vegetation species from a viewpoint of spectral characteristics. So the capability to differentiate the synthetical signal is essential here. Hyperspectral remote sensing is a good tool to implement the differentiation because of its narrow band width. However at present, there only is an on-orbit hyperspectral satellite sensor EO-1 Hyperion with the same spatial resolution of 30 m as Landsat TM, but a fine spectral resolution of about 10 nm. The Hyperion image with a swath of about 7 km needs to be ordered in a schedule time range. Covering all over the Taihu Lake needs ten Hyperion images, which, however, are in different paths with different acquiring time. So it is obvious up to now that the hyperspectral image acquisition is inconvenient and the sensor capability can not fully conform to the requirements of applications.

*Phragmites communi* is mainly regular distributed in the western and southern littoral zone with a range of about 100 m and its biomass is far more than other hydrophytes. According to our experience and previous investigation along the lake shore, there has only been a small change in its spatial distribution since 2001, so in this campaign, *Phragmites communi* was not investigated and the total biomass estimated in this paper doesn't contain of *Phragmites communi*.

It is very interesting to take notice of the phenomenon that the aquatic vegetation is disappearing gradually in some lake zones, such as Gongshan Bay, Xukou Bay and Dongshan Bay; however in other zones it is spreading. The reason why there is an opposite trend for the aquatic vegetation in the same lake is not clear now, which, however, is a very interesting and important problem for scientists in the field to discover change of the Taihu Lake ecosystem.

## 7. Conclusions

It is applicable and practical in technology and methodology for satellite remote sensing to monitor the spatial distribution of aquatic vegetations and their biomass in shallow inland lake. A decision tree with prior knowledge on water transparency may help improve the satellite remote sensing classification accuracy of aquatic vegetation to a certain extent. The empirical model for estimating aquatic vegetation biomass is more accurate and more practical than the semi-analytical model at present. The present empirical model could be conditionally transferred to develop the past model through the relationship between the vegetation index NDVI, respectively, coming from the present and the past satellite imageries.

In Taihu Lake, the total covering area of aquatic vegetation has decreased from 454.6 km^2^ in 2001 to 364.1 km^2^ in 2007; and vegetation in Gongshan Bay has vanished almost entirely, and a great decrease also took place in Xukou Bay and Dongshan Bay. However it is notewrthy that a large spread toward the west along the shore and concurrently toward the north was happening at the same time as the rapid total decrease. At present, the total biomass of aquatic vegetations, excluding *Phragmites communi*, is about 406 (±46.3) thousand tons, mainly distributed in Zhunhu Bay, Guangfu Bay, East Taihu Bay and its mouth zone, those zones joining Xishan island with Dongshan island, and some littoral zones along Qidou-Huzhou. However, it was about 528 (±70.2) thousand tons in 2004, 482 (±67.5) and 489 (±64.4) thousand tons in 2002 and 2001.

## Figures and Tables

**Table 1.** Description of the six clusters and their concomitances.

Vegetation cluster	Main concomitant vegetation
*Potamogeton malaianus* cluster	*Vallisneria natanus*, *Ceratophyllum denersum*, *Hydrilla verticillata*
*Nymphoides peltatum* cluster	*Potamogeton malaianus*, *Trapa incise*, *Vallisneria natanus*
*Nymphoides peltatum*-*Potamogeton malaianus* cluster	*Trapa incise*, *Ceratophyllum demersum*, *Vallisneria natanus*
*Vallisneria natanus* cluster	*Ceratophyllum demersum*, *Elodea nuttalli*, *Hydrilla verticillata*
*Vallisneria natanus*-*Elodea nuttalli* cluster	*Nymphoides peltatum* and *Ceratophyllum demersum*
*Najas marina* cluster	*Potamogeton malaianus*, *Vallisneria natanus*

**Table 2.** The covering area of aquatic vegetation and its biomass in Taihu Lake, no containing *Phragmites communi*; Type one and Type two, respectively, are the floating vegetation-dominated hydrophyte, and the submerged vegetation-dominated hydrophyte.

Date	Type one (km^2^)	Type two (km^2^)	Total area (km^2^)	Total biomass (thousand ton)
15 June 2007	72.4	291.7	364.1	406
26 July 2004	89.1	393.1	482.2	528
13 July 2002	71.7	380.0	451.7	482
26 July 2001	75.8	378.8	454.6	489
